# Axial Length and Ocular Development of Premature Infants without ROP

**DOI:** 10.1155/2017/6823965

**Published:** 2017-10-15

**Authors:** Yi Zha, Guangdong Zhu, Jinfei Zhuang, Haihua Zheng, Jianqiu Cai, Wangqiang Feng

**Affiliations:** The 2nd Affiliated Hospital and Yuying Children's Hospital of Wenzhou Medical University, 109 Xueyuan Road, Wenzhou, Zhejiang 325027, China

## Abstract

**Purpose:**

To investigate the ocular parameters of premature infants without ROP at gestational age (GA) more than 28 weeks and their relationship with growth parameters.

**Methods:**

76 preterm infants without ROP and 65 term infants were involved to undergo portable slit lamp, RetCam3, ultrasonic A-scan biometry, and cycloplegic streak examination at their 40 weeks' postconceptional ages (PCA). Ocular parameters of infants' right eye and growth parameters were used for analysis.

**Results:**

All the infants were examined at 40 weeks' PCA. No significant difference was found between male and female in axial length of preterm infants (*p* = 0.993) and term infants (*p* = 0.591). Significant differences were found in axial length (AL), anterior chamber depth (ACD), lens thickness (LT), and vitreous depth (VD) between preterm and term infants. No significant correlation was found between AL and spherical equivalent in preterm infants' group. In preterm group, AL was significantly correlated with gestational age (GA), birth weight (BW), and head circumference (HC).

**Conclusions:**

Preterm infants had shorter AL, shallow ACD, thicker LT, and thinner VD compared to term infants. Refractive error in preterm infants at GA between 28 to 37 weeks was not related to axial length. Among all the growth parameters of preterm infants, GA, BW, and HC had effect on axial length.

## 1. Introduction

Due to the rapid prevalence of myopia, refractive error has long been the most important eye problem throughout the world especially in Asian countries [1]. Prematurity, with childbirth before 37 weeks of pregnancy, is associated with many ocular abnormalities, such as retinopathy of prematurity (ROP), refractive error, and amblyopia. Refractive error, on the other hand, is found to be related to not only prematurity but also increasing severity of retinopathy of prematurity (ROP) as well [2]. The increasing risk of refractive error in prematurely born infants is nowadays urgent to be solved.

The major proportion of eye growth occurs within the first 12 months after birth [3]. It is well known that term infants are commonly hypermetropic, while preterm infants are always associated with myopia [4–7]. Many researchers have found that the development of myopia in premature infants may be related to ocular parameters, such as axial length and anterior chamber depth [6, 8, 9]. Others tended to believe its association with the corneal curvature and refractive power of the lens [10–12]. Current studies focused more on premature infants with gestational age (GA) lower than 28 weeks, who were apt to suffer from ROP. In the present study, we investigated the ocular parameters of premature infants with GA more than 28 weeks and their relationship with growth parameters.

## 2. Materials and Methods

All the infants involved were recruited from the Department of Neonatology of the 2nd Affiliated Hospital of Wenzhou Medical University. Regional ethics committee approval and parental consent were obtained. Exclusion criteria included ocular anomalies such as microphthalmos, anophthalmos, craniofacial deformities, congenital glaucoma, cornea, and lens, or any other fundus abnormalities (such as ROP) and a history of cerebral damage. Premature infants with GA of less than 28 weeks were also excluded in the study. There was no geographic or ethnic dissimilarity in this study. All the infants were Chinese.

At the first visit, growth parameters such as GA, length, birth weight (BW), and head circumference (HC) at birth were obtained. At 40 weeks' postconceptional ages (PCA) (defined as the gestational age at birth plus the age in weeks at the time of examination), all the infants including premature and term infants underwent portable slit lamp, RetCam3 (Clarity Medical System, USA), ultrasonic A-scan biometry, and cycloplegic streak retinoscopy (cyclopentolate 0.5%) instilled at 10-minute intervals three times, 40 minutes before retinoscopy. RetCam3 was used to exclude ROP or other retinal disease. Cycloplegic streak retinoscopy was used to get the refractive status of each infant. The ultrasonic examination was performed using an A-scan biometer (Carl Zeiss Meditec, Oberkochen, Germany) to measure the axial length (AL), anterior chamber depth (ACD), lens thickness (LT), and vitreous depth (VD). The probe was placed lightly on the center of the cornea, perpendicular to its axis. The probe was maintained in this position until three clear traces were obtained on the screen. The average value from the three images was recorded.

All the data obtained were analyzed using SPSS (version 17.0; SPSS Inc., Chicago, IL, USA). The values obtained for all parameters were expressed as mean ± SD. *t*-test was used to compare parameters between two groups. Simple linear regression analysis was performed to get the association of axial length with each of the variables, namely, spherical equivalent (SE), GA, length, BW, and HC. A *p* value below 0.05 was considered significant.

## 3. Results

76 premature infants (with 27 female and 49 male) and 65 term infants (with 32 female and 33 male) were involved in the research. The data of infants' right eye were chosen for analysis. The GA in the preterm group was 32.97 ± 2.15 w and in the term group, 39.29 ± 1.30 w. All the growth parameters for preterm and term infants were shown in Table 1. No significant difference was found between male and female in axial length of preterm infants (*p* = 0.993) and term infants (*p* = 0.591). The mean and SD of infants' SE, AL, ACD, LT, and VD were shown in Table 2. Significant differences were found in AL, ACD, LT, and VD between preterm and term infants' groups.

In preterm group, AL was significantly correlated with GA (*r* = 0.312, *p* = 0.006), BW (*r* = 0.344, *p* = 0.002), and HC (*r* = 0.241, *p* = 0.041) but not correlated with SE (*r* = −0.226, *p* = 0.05) and length (*r* = 0.229, *p* = 0.053).

## 4. Discussion

This research focused on Chinese premature infants born at GA between 28 to 37 weeks without ROP or other ocular disease and evaluated the association of AL with other ocular and growth parameters. Our study showed that with a GA of 28 to 37 weeks, premature infants did have lower AL, shallow ACD, thicker lens, and shorter VD (Table 2). AL was significantly correlated with GA, BW, and HC.

Fieß A et al. [13] once compared the axial length and anterior segment alterations in preterm infants with those of full-term infants and found significant differences between preterm and full-term infants aged ≤ 7 years for spherical equivalent, astigmatism, corneal diameter, and axial length. Tian et al. [14] investigated the development of the refractive status in premature infants aged 0 to 6 years old and found that the axial length in preterm infants was significant shorter than that in term infants. Ecsedy et al. [15] compared the ocular geometry and refraction in children with a history of preterm birth and found that in the premature eyes, anterior chamber depth was marginally smaller, the lens was significantly thicker, and axial length was significantly shorter. All the research above focused on older preterm children. In our research, we measured the ocular parameters in preterm infants with GA between 28 and 37 weeks without ROP and found that preterm infant had shorter AL, smaller ACD, thicker lens, and shorter VD. As we know, ocular structures go through a continuous development and remodeling process after birth [16]. Premature departure from the intrauterine environment may affect ocular development [17]. We believe that premature birth even with GA older than 28 weeks delays the development of ocular structures, which happens sooner after birth.

Preterm infants were thought to be more myopic than term infants [8]. However, ocular biometry and the mechanism of myopia in preterm children were somewhat different from those in full-term children. Anterior segment components were thought to contribute more to myopia progression in preterm children [8, 18–20]. Axial length, on the other hand, changed differently [18–21]. According to our result, axial length had no significant relationship with spherical equivalent. It was considered that early refractive errors may not provide enough information in predicting the later refractive and axial length outcome.

Fieß A et al. [13] detected axial length associated with birth weight and age. Modrzejewska et al. [9] found a correlation between axial length and birth weight. In our research, AL was significantly correlated with GA, BW, and HC. As the major proportion of eye growth occurs within the first year after birth, our observation suggested that ocular growth be affected by prematurity and led to shorter axial length, especially in the first years of life.

The prematurely born children had a higher prevalence of hypermetropia (>3 D) and clinically significant myopia (≤−1 D) than those born at term. Moderate or high myopia (<−3 D) was found only in preterm group. In our preterm group, take the right eye for granted, there were 22.37% (17/76) of hypermetropia (>3 D) and 15.78% (12/76) of significant myopia (≤−1 D). Four infants had moderate or high myopia (≤−3 D). The percentages of hypermetropia and significant myopia were higher than those of our term group (with 10/65 of hypermetropia and 5/65 of significant myopia). Due to the limitation of sample size, further studies were needed for larger sample investigation.

In this research, we investigated the axial length and refractive status of preterm infants with a GA of more than 28 weeks without ROP and found that preterm infants had shorter AL, shallow ACD, thicker LT, and thinner VD. Axial length in preterm infants was significantly correlated with gestational age, birth weight, and head circumference.

## Figures and Tables

**Table 1:** Growth parameters and age for both preterm and term infants.

Growth parameters	Preterm infants (mean ± SD)	Term infants (mean ± SD)
Gestational age (weeks)	32.97 ± 2.15	39.29 ± 1.30
Birth weight (g)	1930 ± 525	3232 ± 475
Head circum. (cm)	30.68 ± 1.98	33.93 ± 1.35
Length (cm)	42.83 ± 4.38	49.98 ± 2.50

**Table 2:** Parameter analysis of variance between preterm and term infants at 40 weeks' PCA.

Parameter for right eye	Term infants (mean ± SD)	Preterm infants (mean ± SD)	*p*
Number	65	76	
Spherical equivalent (SE, D)	2.19 ± 2.22	1.96 ± 2.12	0.50
Axial length (AL, mm)	17.34 ± 0.55	17.08 ± 0.67	^∗^ **0.02**
Anterior chamber depth (ACD, mm)	2.55 ± 0.26	2.38 ± 0.25	^∗^ **0.000**
Lens thickness (LT, mm)	3.72 ± 0.18	3.99 ± 0.15	^∗^ **0.000**
Vitreous depth (VD, cm)	11.03 ± 0.46	10.79 ± 0.56	^∗^ **0.01**

$\begin{array}{c} {}^{*}p<0.05 \end{array}$ significant difference between two groups.
